# Hospitals and the Truck Act

**Published:** 1914-01-24

**Authors:** Alfred Whitham

**Affiliations:** Beckett Hospital, Barnsley


*To the Editor of* The Hospital.

### Hospitals and the Truck Act.

*To the Editor of* The Hospital.

Sir,—With your permission may I draw the attention of the British Hospitals Association to a Bill now before Parliament to amend and extend the Truck Acts, 1830 to 1896? The amending Bill was read a first time in the House of Commons on July 30, 1913, backed by Lord Henry Cavendish Bentinck, Mr. Noel Buxton, Mr. O'Grady, Mr. Rowntree, and Mr. Stephen Walsh.

The object of the Bill is to amend the Truck Acts by making illegal all deductions from wages and all fines, and particularly states in Clause 1, Section 1 (*e*), that "an employer shall not make any deduction or require any payment in respect of any subscription or payment to, or otherwise in connection with, any benefit society, sick club, hospital, or other society, club, or benefit, or for medical attendance."

If this clause is passed as printed a serious blow will be dealt to the hospitals in the Midland and Northern Counties. Up to the present time it has been the custom of workmen, with the sanction of the owners of mines, to allow weekly, monthly, or yearly payments to be deducted from their wages in aid of the hospitals in their district and the gross amount handed over in one lump sum. If this system is stopped through the action of the said Bill then the income to hospitals will not only be decreased, but the cost of collection would be so great that a very poor net result would accrue.

It has already been pointed out to the Home Secretary that practically in all Parliamentary proceedings with regard to truck since 1831 it has generally been admitted to have been satisfactorily shown that provident institutions, such as miners' permanent relief societies, together with hospitals, might fairly be held to be purely benevolent agencies and consequently separate and distinct from the idea usually associated with reference to truck.

There is every probability that the Bill will again be before Parliament during next Session, and something ought to be done to claim exemption as far as our hospitals are concerned, or another serious blow will be given to voluntary hospitals, especially to those in the Northern district. A very strong case might be made out on their behalf claiming exemption from the proposed Bill.—Yours obediently, Alfred Whitham.

Beckett Hospital, Barnsley.

